# *Chrysosporium* sp. Infection in Eastern Massasauga Rattlesnakes

**DOI:** 10.3201/eid1712.110240

**Published:** 2011-12

**Authors:** Matthew C. Allender, Michael Dreslik, Sarah Wylie, Christopher Phillips, Daniel B. Wylie, Carol Maddox, Martha A. Delaney, Michael J. Kinsel

**Affiliations:** University of Illinois, Urbana, Illinois, USA

**Keywords:** zoonoses, fungal infections, fungi, reptiles, eastern massasauga rattlesnake, Sistrurus catenatus catenatus, Chrysosporium

**To the Editor**: During 2008, the ninth year of a long-term biologic monitoring program, 3 eastern massasauga rattlesnakes (*Sistrurus catenatus catenatus*) with severe facial swelling and disfiguration died within 3 weeks after discovery near Carlyle, Illinois, USA. In spring 2010, a similar syndrome was diagnosed in a fourth massasauga; this snake continues to be treated with thermal and nutritional support and antifungal therapy. A keratinophilic fungal infection caused by *Chrysosporium* sp. was diagnosed after physical examination, histopathologic analysis, and PCR in all 4 snakes. The prevalence of clinical signs consistent with *Chrysosporium* sp. infection during 2000–2007 was 0.0%, and prevalence of *Chrysosporium* sp.–associated disease was 4.4% (95% confidence interval [CI] 1.1%–13.2%) for 2008 and 1.8% (95% CI 0.0%–11.1%) for 2010.

Clinical and gross necropsy abnormalities were limited to the heads of affected animals. In each case, a unilateral subcutaneous swelling completely obstructed the nasolabial pits (Figure, panel A). In the most severely affected snake, swelling extended to the cranial aspect of the orbit and maxillary fang (Figure, panel B). Notable histologic lesions were restricted to skin, gingiva, and deeper tissues of the head and cervical region and consisted of cutaneous ulcers with granulomas in deeper tissues (Figure, panel C). Ulcers had thick adherent serocellular crusts and were delineated by small dermal accumulations of heterophils and fewer macrophages. Crusts contained numerous 4–6-µm diameter right-angle branching fungal hyphae with terminal structures consistent with spores. In 1 snake, infection was associated with retained devitalized layers of epidermis consistent with dysecdysis. In the same snake, the eye and ventral periocular tissues were effaced by inflammation, but the spectacle and a small fragment of cornea remained; the corneal remnant contained few fungal hyphae.

**Figure:**
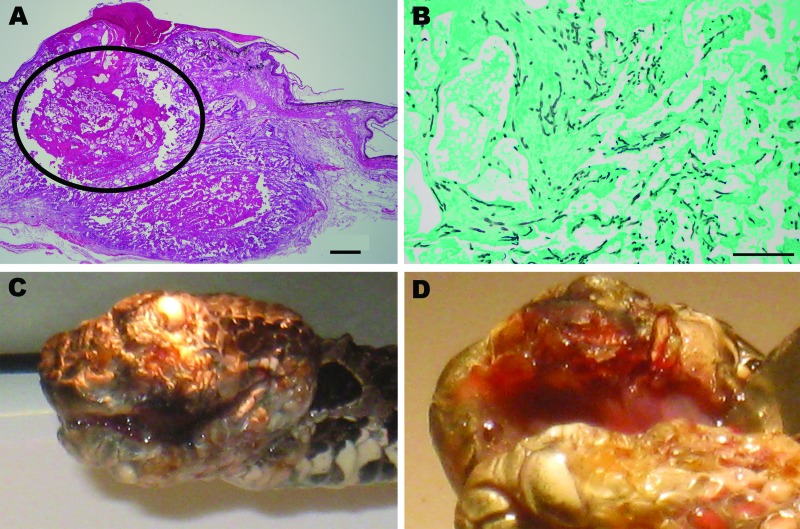
*Chrysosporium* sp. fungal infection in eastern massasagauga rattlesnake (*Sistrurus catenatus catenatus*). A) Facial dermatitis and cellulitis caused by *Chrysosporium* sp. infection in rattlesnake from Carlyle, Illinois, USA; B) close-up showing maxillary fang destruction. C) Maxillary dermal and subcutaneous fungal granuloma (circled area). Hematoxylin and eosin stain, original magnification ×2, scale bar = 500 μm. D) Granuloma center with large numbers of fungal hyphae. Grocott methenamine silver stain, original magnification ×10, scale bar = 100 μm.

In all snakes, in addition to deep cutaneous ulceration, the dermis, hypodermis and skeletal muscle of the maxillary and or mandibular region contained multiple granulomas, centered on variable numbers of fungal hyphae (Figure, panel D). In 1 snake, similar granulomas were also observed in maxillary gingival submucosa and subjacent maxillary bone.

Five frozen skin biopsy samples from 4 snakes were thawed and plated on Sabaroud agar; however, no fungal growth was recovered. Genomic DNA was extracted from tissue, and PCR was performed by using 2 sets of fungus-directed rRNA gene primers. The DNA was sequenced, and the 4 amplicons showed >99% homology with *C. ophiodiicola* (GenBank accession no. EU715819.1).

Fungal pathogens have been increasingly associated with free-ranging epidemics in wildlife, including the well-known effects of *Batrachochytrium dendrobatidis* on frog populations globally (*1*) and white-nose syndrome in bats (*2*). Both of these diseases cause widespread and ongoing deaths in these populations that seriously threaten biodiversity across the United States (*1**,**2*). Furthermore, the emergence of keratinophilic fungi, *Chrysosporium* anamorph *Nannizziopsis vriesii*, caused fatal disease in captive bearded dragons within the past decade (*3**,**4*). Keratinophilic fungi have received considerable interest recently because of pulmonary or dermatologic disease caused in immunocompromised humans and prevalence in hospitals (*5**,**6*).

The *Chrysosporium* sp. fungi recently identified in the snakes from the Carlyle Lake area is molecularly related to a *Chrysosporium* sp. from diseased skin in a captive snake (*7*). Fungal diseases in reptiles are commonly secondary or opportunistic pathogens. However, *Chrysosporium* anamorph *Nannizziopsis vriesii* (*3**,**4**,**8*) and the *Chrysosporium* sp. reported here in massasaugas are occurring in animals as primary pathogens.

We describe evidence of *Chrysosporium* sp. causing death in free-ranging snakes. To our knowledge, this is the first reported occurrence of any similar disease syndrome in this population. Before 2008, these clinical signs had not been witnessed during radiotelemetry and mark-recapture studies or in health monitoring studies (*9**,**10*). More intensive health monitoring programs are warranted at this site, as well across this species’ range. Whether this disease represents isolated emerging incidents in Illinois or indicates more widespread concern for this species, as has been documented in bats with white-nose syndrome (*2*), is unclear.

Origin, transmission, and treatment of *Chrysosporium* sp. are unknown. The eastern massasaugas in this investigation carrying the fungal infection were from 2 discontiguous sites; therefore, direct transmission is not necessary. The occurrence across different locations and in different years suggests the organism is present in the environment, and histopathologic results indicative of primary skin involvement were consistent with environmental acquisition of infection. Potential causes for the development of lesions specifically to the head include primary trauma, high local environmental load, or disruption of the normal skin defense mechanisms.

This fungal pathogen has serious long-term implications for this population of endangered snakes. There is no indication that hikers in this environment are at risk, but continued monitoring of human and wildlife health is essential to assess environmental and zoonotic disease risks. Furthermore, if human behavior can alter disease transmission (e.g., through hiking behaviors), disease prevention at Carlyle Lake, which hosts >1 million visitors annually, will likely be unsuccessful.
